# Evaluation of two different *Cannabis sativa* L. extracts as antioxidant and neuroprotective agents

**DOI:** 10.3389/fphar.2022.1009868

**Published:** 2022-09-13

**Authors:** Guillermo Cásedas, Cristina Moliner, Filippo Maggi, Eugenia Mazzara, Víctor López

**Affiliations:** ^1^ Department of Pharmacy, Faculty of Health Sciences, Universidad San Jorge, Zaragoza, Spain; ^2^ Instituto Agroalimentario de Aragón-IA2 (CITA-Universidad de Zaragoza), Zaragoza, Spain; ^3^ School of Pharmacy, University of Camerino, Camerino, Italy

**Keywords:** trans-Δ-9-tetrahydrocannabinol (Δ9-THC), cannabidiol (CBD), cannabinoid, polyphenol, oxidative stress, antioxidant, enzymatic inhibition, central nervous system (CNS)

## Abstract

*Cannabis sativa* L. is a plant that contains numerous chemically active compounds including cannabinoids such as trans-Δ-9-tetrahydrocannabinol (Δ9-THC) and cannabidiol (CBD), and flavone derivatives, such as luteolin-7-O-glucuronide and apigenin glucuronide. In particular, the polar fraction of hemp including many phenolic compounds has been overlooked when compared with the more lipophilic fraction containing cannabinoids. Therefore, the aim of this study was to assess two extracts of industrial hemp (*C. sativa*) of different polarity (aqueous and hexane) by evaluating their antioxidant profile and their neuroprotective potential on pharmacological targets in the central nervous system (CNS). Several assays on *in vitro* antioxidant capacity (DPPH, superoxide radical, FRAP, ORAC), as well as inhibition of physiological enzymes such as acetylcholinesterase (AChE) and monoaminooxidase A (MAO-A) were carried out in order to find out how these extracts may be helpful to prevent neurodegenerative disorders. Neuro-2a cell line was selected to test the cytotoxic and neuroprotective potential of these extracts. Both extracts showed striking antioxidant capacity in the FRAP and ORAC assays, particularly the hexane extract, and interesting results for the DPPH and superoxide radical uptake assays, with the aqueous extract standing out especially in the latter. In enzyme inhibition assays, the aqueous extract showed AChE and MAO-A inhibitory activity, while the hexane extract only reached IC_50_ value for AChE inhibitory bioassay. Neuro-2a assays demonstrated that polyphenolic extract was not cytotoxic and exhibited cytoprotective properties against hydrogen peroxide and antioxidant response decreasing reactive oxygen species (ROS) production. These extracts could be a source of compounds with potential benefit on human health, especially related to neurodegenerative disorders.

## 1 Introduction

*Cannabis sativa* L. (Cannabaceae) is an annual herbaceous plant native to Central Asia (China and India) but cultivated all over the world. Since antiquity it has been used as a source of fiber, food, oil and medicine, as well as for recreational and religious purposes (Russo et al., 2008). *C. sativa* contains a series of chemically active compounds such as cannabinoids, terpenes, flavonoids and alkaloids (Andre et al., 2016). The most active are cannabinoids, a class of terpene-phenolic compounds, accumulated in the trichome cavity of female flowers. Over 100 cannabinoids have been identified, the most powerful is trans-Δ-9-tetrahydrocannabinol (Δ9-THC), which is predominantly responsible for the psychotropic effects (Whiting et al., 2015), thus being minoritarian (<0.2% in the biomass dw) respect to cannabidiol (CBD) in the industrial hemp varieties.

Flavonoids are a group of phenolic compounds and therefore water-soluble compounds from plants with proven beneficial effects on several common diseases including chronic inflammation, cancer, neurodegenerative disorders, cardiovascular complications and hypoglycaemia (Cermak, 2007; Chen and Luo, 2010). Flavonoids are classified according to their structural variations, the most notable being flavones, flavonols, flavanones, anthocyanins and chalcones (Peñarrieta et al., 2014). The main flavonoids found in *C. sativa* are flavone glycosides among which luteolin-7-O-glucuronide and apigenin glucuronide are the most representative compounds (Bautista et al., 2021).

In contrast, phytocannabinoids are lipophilic compounds and are classified into neutral cannabinoids (with no carboxyl group) and cannabinoid acids (with a carboxyl group). In *C. sativa*, cannabinoids are biosynthesized and accumulated as cannabinoid acids and subsequently decarboxylated to their neutral forms. In this manner, cannabidiolic acid (CBDA) and Δ-9-tetrahydrocannabinolic acid (Δ9-THCA) are obtained from cannabigerolic acid (CBGA), from which the neutral forms such as cannabidiol (CBD) and Δ9-THC are produced by decarboxylation (Sirikantaramas and Taura, 2017).

Despite the structural similarity between CBD and Δ9-THC, CBD has low agonism for cannabinoid receptors. Current evidence shows that CBD is endowed with anticonvulsant, antispasmodic, anxiolytic, anti-nausea, anti-rheumatoid arthritis, and neuroprotective properties (Ibeas Bih et al., 2015). Recently, CBD has been shown to be an inverse agonist of orphan G-protein coupled receptors such as GPR3, GPR6 and GPR12, suggesting new therapeutic uses of CBD for Alzheimer’s disease, Parkinson’s disease, cancer and infertility (Laun et al., 2018).

Phytocannabinoids and polyphenols from *C. sativa* have shown to affect redox balance by modifying the level and activity of oxidants and antioxidants (Pellati et al., 2018). In the same way as other antioxidants, CBD, Δ9-THC and flavonoids of the above-mentioned plant interrupt the chain reactions of free radicals, neutralizing them or transforming them into less active forms. Furthermore, they reduce oxidative conditions by preventing the formation of superoxide radicals, which are mainly generated by xanthine oxidase (XO), and also reduce the production of reactive oxygen species (ROS) by chelating transition metal ions (Pollastro et al., 2017; Atalay et al., 2019).

Oxidative stress has been associated with chronic inflammation in neurodegenerative diseases such as Alzheimer’s, Parkinson’s disease and amyotrophic lateral sclerosis (ALS), with an increasing number of elderly individuals suffering from it (Peng et al., 2020). The brain is especially vulnerable to oxidative stress and oxidative damage, due to its high oxygen consumption, its low antioxidant defences, and its high content of polyunsaturated fats that are very prone to oxidation (Rodriguez-Martin et al., 2019).

CBD has antioxidant properties that depend on its chemical structure, showing neuroprotective effects by decreasing oxidative parameters and increasing cell viability. According to Campos et al. (2016), it has been observed that, in patients with Alzheimer’s or multiple sclerosis who have been pre-treated with CBD, the accumulation of ROS, lipid peroxidation, caspase-3 levels and DNA fragmentation have been reduced in stimulated PC12 cells. Some studies suggest that Δ9-THC also has neuroprotective properties because it reduces the decrease in dopamine content and tyrosine hydroxylase activity by inhibiting oxidative stress (Junior et al., 2019).

Another study on PC12 cells and SH-SY5Y neurons subjected to 6-OHDA and supplemented with polyphenols such as protocatechuic acid (PCA), gallic acid, cyanidin and delphinidin metabolites, demonstrated an increase in the activity and expression of antioxidant proteins regulated by Nrf2 (Zhang et al., 2015; Chandrasekhar et al., 2018). Furthermore, luteolin is a neuroprotective flavonoid that inhibits nitric oxide (NO), hydrogen peroxide (H_2_O_2_) and malondialdehyde (MDA), and normalizes the activities of superoxide dismutase (SOD), acetylcholinesterase (AChE), and glutathione S-transferase in rats (Akinrinde and Adebiyi, 2019). Additionally, apigenin has been reported as a natural anti-carcinogenic, anti-inflammatory and antioxidant scavenger against oxidative stress, it also increased intracellular antioxidant defences as well as it has been studied for Alzheimer’s disease model as a potential drug in mice (Nabavi et al., 2018).

Due to the benefits that different compounds from *C. sativa* (mainly CBD and Δ9-THC) generate, in recent months the idea of producing medicines or food supplements based on cannabinoid compounds has been greatly reinforced. That is why the approval of some cannabinoids by the Food and Drug Administration (FDA) has opened the horizon for the use of these natural drugs as medicines. In June 2018, the FDA approved the first CBD drug called Epidiolex^®^, useful for the therapy of refractory epilepsy (ERT), including Lennox-Gastaut syndrome (LGS) and Dravet syndrome (SD), and Sativex^®^, a drug based on THC and CBD, used for the treatment of spasticity caused by multiple sclerosis (Li et al., 2020).

On the other hand, less is known on the pharmacological potential of the more polar fraction of *C. sativa* extract where the major constituents are flavone glycosides. For this reason, the aim of this study is to clarify the antioxidant properties of two extracts of *C. sativa*; the lipophilic (hexanic) fraction is vastly composed of phytocannabinoids, while the hydrophilic (aqueous) fraction contains mainly polyphenols such as flavones. The pharmacological role of these extracts as neuroprotective agents can be considered after these encouraging results.

## 2 Material and methods

### 2.1 Chemicals and reagents

Neuro-2a (N2a) cell line was acquired from the Collection (ATCC, Manassas, VA, United States) whereas 3-(4,5-Dimethyl-2-thiazolyl)-2,5-diphenyltetrazolium bromide (MTT), bovine serum albumin (BSA), penicillin, streptomycin, 2,2-diphenyl-1-picrylhydrazyl (DPPH), Trolox, Monoamine oxidase A (MAO-A), Trizma base, 4-aminoantipyrine, 5,5′-dithiobis-(2-nitrobenzoic acid) (DTNB), tyramine, levodopa (L-DOPA), horseradish peroxidase, 2,2′-azobis (2-methyl-propionamidine)-di-hydrochloride (AAPH), hydrogen peroxide (30%), vanillic acid, tyrosinase, 2,4,6-Tris (2-pyridyl)-1,3,5-triazine (TPTZ), galantamine, 2′,7′-Dichlorodihydrofluorescein Diacetate (DCFH-DA), xanthine, ferrous sulfate (FeSO_4_), acetylcholinesterase (AChE), acetylthiocholine iodide (ATCI) were obtained from Sigma-Aldrich (Madrid, Spain). Clorgyline and α-kojic were from Cymit química (Barcelona, Spain). Sodium carbonate (Na_2_CO_3_), HCl, NaCl, dimethyl sulfoxide (DMSO), methanol, and potassium phosphate were supplied from Panreac (Barcelona, Spain). Dulbecco’s modified Eagle’s medium (DMEM), fetal bovine serum (FBS), Nitroblue Tetrazolium (NBT) and xanthine oxidase were purchased from Vidrafoc (Barcelona, Spain) and iron chloride (FeCl_3_) from Laboaragon (Zaragoza, Spain). All reagents used were of analytical quality.

### 2.2 Plant material and extraction process

Futura 75 hemp frozen inflorescences, cultivated in Fiuminata (central Italy; 43° 10′ 40″ N, 12° 56′ 59″ E; 451 m asl), were employed to obtain the extracts by microwave-assisted extraction (MAE) using a Milestone ETHOS X system (Milestone, Italy) (Mazzara et al., 2022). The process was run by applying a microwave power of 1.5 W/g, an extraction time of 160.5 min and a percentage of added water into the reactor of 55.4%. The two products, namely the aqueous extract and residual biomass, were collected from the reactor to be analysed. Before characterization, the aqueous extract was filtered, stored at −20°C, and then freeze dried with a BUCHI Lyovapor L-200 (Büchi Labortechnik AG, Flawil, Switzerland) at −54°C, 10°C shelf temperature and 0.05 mbar pressure. The obtained lyophilized aqueous extract (AE) was milled using a mortar and pestle and the powder was kept at 4°C until analysis. On the other hand, the residual biomass was dried for 24 h at 60°C in a ventilated oven (Biosec, Tauro Essiccatori, Vanzo Nuovo, Vicenza, Italy), and stored at room temperature in the dark. Then, it was powdered using a MFC, IKA-Werk (Staufen, Germany), then extracted with analytical-grade *n*-hexane (Sigma-Aldrich) in an ultrasound bath (AU-220, Argo Lab, Carpi, Italy) at room temperature for 20 min, in order to obtain the residual biomass hexane extract (HE).

### 2.3 Neurodegenerative enzymes

A wide range of concentrations of both hemp extracts was selected to inhibit monoamine oxidase A (MAO-A) and acetylcholinesterase (AChE), which are CNS related enzymes with important pharmacological applications.

#### 2.3.1 Acetylcholinesterase inhibition assay

The samples were then assayed for AChE inhibition using the Ellman’s method with slightly adjustments (Rhee et al., 2001). Each well contained a mix of ATCI (15 mM), DTNB (3 mM) in a Tris-HCl buffer (50 mM, pH = 8); BSA and selected range of concentrations for samples or buffer (control). After all, AChE enzyme (0.22 U L^−1^) was combined to begin the reaction. Galantamine was employed as the reference drug. Optical density was read 13 times at 405 nm in a 96-well plate.

#### 2.3.2 Monoamine oxidase-A inhibition assay

Hemp extracts were analysed for MAO-A inhibition as previously reported by Olsen and collab (Olsen et al., 2008). Each well contained designated range of concentrations for hemp extracts or buffer (control), chromogenic solution (0.8 mM vanillic acid, 417 mM 4-aminoantipyrine and horseradish peroxidase (4 U mL^−1^) in potassium phosphate buffer pH = 7.6), tyramine (3 mM) and MAO-A (8 U mL^−1^). Clorgiline was employed as the reference drug. The absorbance was read at 490 nm every 5 min for 30 min in a FLUOstar Omega microplate reader (BMG Labtech, Ortenberg, Germany).

### 2.4 Antioxidant profile

Antioxidant capacity was assessed by four complementary methods that were DPPH, superoxide radical, FRAP and ORAC.

### 2.4.1 DPPH scavenging capacity

The neutralization of DPPH radicals was conducted according to the modifications described by López et al. (2007). In a 96-well plate the same parts of extracts and DPPH solutions (0.04 mg/ml in methanol) were mixed. Antiradical activity was detected at 516 nm after 30 min of dark incubation at room temperature. Ascorbic acid was used as a positive standard.

#### 2.4.2 Superoxide radical reduction by xanthine/xanthine oxidase system

The substrate xanthine and the enzyme xanthine oxidase can be used to measure the potential reduction of superoxide radical by the formation of the NBT-radical superoxide complex. As well as DPPH assay, several dilutions were prepared to establish IC_50_ values. The assay was carried out in a 96-well microplate and contained a mix of diluted concentrations (30–1,000 μg/ml) of hemp extracts/buffer, Na_2_CO_3_ (16 mM), NBT (22.8 µM), xanthine (90 µM) and xanthine oxidase (0.168 U mL^−1^). Ascorbic acid was employed as a positive standard. Then, the mix reaction was incubated for 2 min at 37°C and absorbance was read at 560 nm (Rodríguez-Chávez et al., 2015).

#### 2.4.3 Ferric reducing antioxidant power (FRAP)

FRAP assay was conducted to analyse antioxidant properties of hemp extracts. The reaction measures reduction of TPTZ to a blue product using FeCl_3_ and buffer solution at 595 nm (Fernández-Moriano et al., 2016). Hemp extracts were mixed with fresh daily FRAP reagent and incubated 30 min at 37°C. FeSO_4_ was used as standard to express results as µmol Fe^2+^/g extract.

#### 2.4.4 Oxygen radical absorbance capacity (ORAC)

ORAC assay established the capacity of hemp extracts to scavenge peroxyl radicals. The protocol was adapted from Dávalos et al. (2004) and trolox (100 µM) was used as antioxidant reference compound. Samples were incubated with fluorescein (116 nM) for 10 min at 37°C in a dark plate. Then, AAPH (40 mM) was added to the wells and loss of fluorescence was measured at excitation and emission wavelengths of 485 nm and 520 nm, respectively. The spectrophotometer for this assay was Synergy H1 BioTek and results were expressed as µmol Trolox equivalents (TE)/mg sample.

### 2.5 Polyphenolic extract activity on Neuro-2a cells

#### 2.5.1 Cell culture

Neuro-2a cells (ATCC^®^ CCL-131) were defrosted and cultured in 10% FBS-supplemented high-glucose DMEM and 1% penicillin-streptomycin, seeded in a T25 flask, and placed into the incubator (5% CO_2_, 37°C). The experiments took place between passages 12–18. Density for a 96-well plate was 1 × 10^4^ cells/well to carry out each assay.

#### 2.5.2 Treatment solutions

Stock solutions of AE (1.000 μg/ml) were prepared in sterilized phosphate buffered saline (PBS). The sample was vortexed and filtered with a 0.22-µm syringe filter. Hydrogen peroxide (1,000 µM) was prepared daily and combined into the cells at a final concentration of 300 µM (1 h) inciting oxidative stress to the cells.

#### 2.5.3 MTT assay

Mitochondrial response was evaluated by MTT assay. First, the cytotoxicity of the extract was measured in a concentration range (25–1,000 μg/ml). Moreover, the cytoprotective properties of polyphenolic (AE) and cannabinoid (HE) extracts were established on Neuro-2a running again in a different condition by using hydrogen peroxide (300 µM) as a neurotoxic agent inducing oxidative stress for 1 h. After that time, the prooxidant was removed and cells were cultured for 24 h in 1% FBS-supplemented high-glucose DMEM. After treatments, MTT solution was added to the cells, after formazan crystals were dissolved, absorbance was measured at 550 nm using a Synergy H1 BioTek microplate reader (Agilent Technologies, Madrid, Spain). Results were expressed as percentage of control and experiments were performed at least four times.

#### 2.5.4 ROS production

Neuro-2a cells were treated with different conditions of hemp AE and HE, and hydrogen peroxide simultaneously. Intracellular reactive oxygen species (ROS) were revealed by using DCFH-DA as a fluorescent probe. The experiment was carried out following the protocol developed by Lebel and collab (LeBel et al., 1992), with some modifications. ROS generation was measured at 480 nm of excitation and 510 nm of emission at 37°C for 90 min.

#### 2.5.5 Microscopy

Direct visual observation of the treated cells for any morphological changes or toxicity was performed under an inverted microscope (Leica DMi1, 40x and 100x). Photos were taken with Leica Las X software.

### 2.6 Statistical analysis

Minimum three independent biological replicates (i.e., independent passages in independent experiments/stimulations) were performed for all experiments in three different days. The bar graphs depict mean +SEM or 95% CI. Non-linear regression was selected to calculate IC_50_ inhibitory assays. Groups were compared via Student’s t-test or ANOVA followed by Dunnett’s multiple comparison test using GraphPad Prism 8 software as indicated in the figure legends. Differences considered as significant (*p* < 0.05) are labelled with asterisk (∗) within graphs.

## 3 Results

### 3.1 Extracts composition

AE and HE chemical composition are reported in Mazzara et al. (2022). HPLC-DAD-MS^*n*^ analysis performed on AE and HE revealed a significant content of several bioactive substances, that could be exploited on an industrial level, especially in the pharmaceutical and nutraceutical fields. In particular, AE was found to be rich in phenolic derivatives and, among them, luteolin-7-O-glucuronide and apigenin glucuronide were the main ones (31.1 mg/g and 15.4 mg/g, respectively). The cannabinoids fraction in AE was almost missing, in fact only CBD was detected, and in low amount (0.85 mg/g) (Mazzara et al., 2022). On the other hand, HE presented an interesting cannabinoids profile, especially in the decarboxylated form, with CBD as the predominant one (160.5 mg/g). Notably, the extraction procedure didn’t lead to a complete decarboxylation of cannabinoid acids, as demonstrated by the presence of some of them in HE, such as CBDA (14.4 mg/g). Consequently, this study showed that AE could be exploited as a source of glycosidic flavones, while HE could be used to obtain phytocannabinoids.

### 3.2 Neurodegenerative enzymes

**FIGURE 1:**
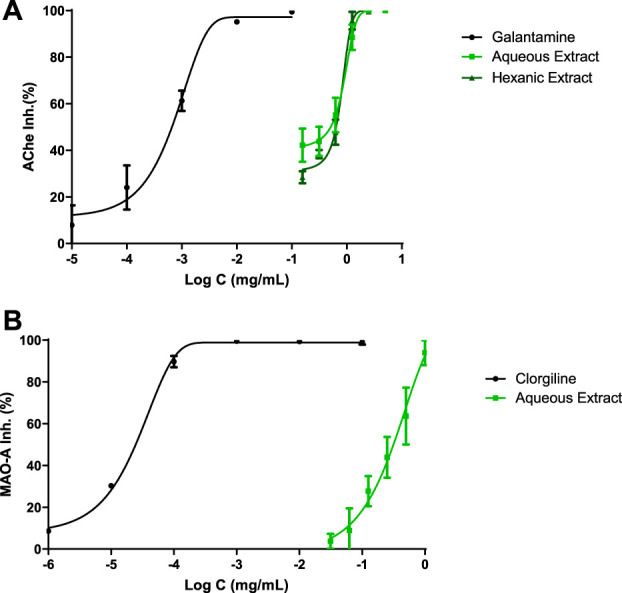
CNS enzyme inhibitions. IC_50_ values were calculated by non-linear regression. **(A)** Acetylcholinesterase inhibition profiles of Cannabis extracts and galantamine. **(B)** Monoamine oxidase A (MAO-A) inhibition profiles of polyphenolic Cannabis extract and clorgiline.

Figure 1 shows the inhibitory capacity of hemp extracts on CNS enzymes. Both extracts were capable of inhibiting AChE enzyme while HE could not reach IC_50_ value for MAO-A enzyme at tested concentrations.

What can be seen in this graph (Figure 1A) is that every tested compound reached 100% of AChE inhibition. Clearly, galantamine showed a greater IC_50_ meaning in 1 μg ml^−1^, while AE and HE showed IC_50_ values of 502 and 640 μg ml^−1^, respectively.

MAO-A inhibition was analyzed in Figure 1B; clorgiline, which was the reference drug, exhibited 0.02 μg ml^−1^ as IC_50_ value. On the other hand, AE displayed a value of 315 μg ml^−1^ and achieved the total inhibition of the enzyme.

### 3.3 Antioxidant profile

In order to determine the antioxidant profile of the extracts, four different methods have been used such as DPPH and superoxide radical scavenging, FRAP and ORAC assays.

**FIGURE 2:**
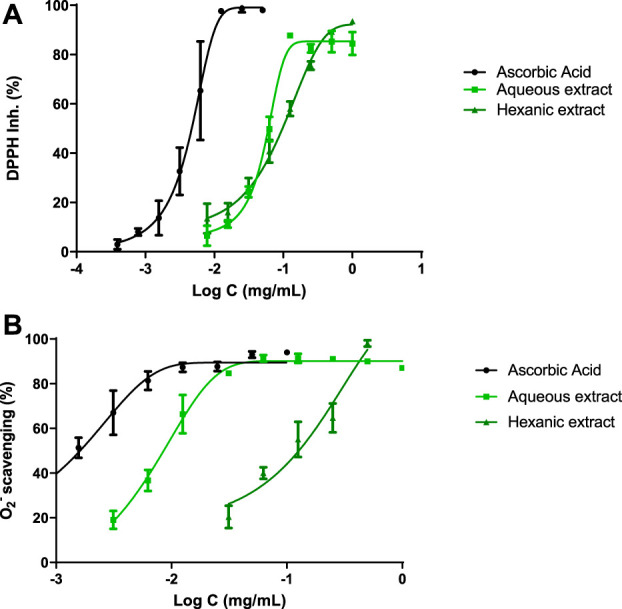
Antioxidant profile. IC_50_ were calculated by non-linear regression. **(A)** DPPH inhibition of hemp extracts. **(B)** Hemp extracts scavenge superoxide radicals generated by the xanthine/xanthine oxidase system. Ascorbic acid was used as reference antioxidant.

Figure 2A shows the DPPH scavenging capacity of both hemp extracts and ascorbic acid as reference compound. A non-linear regression was displayed and IC_50_ values were achieved for each compound. These values were 5 μg ml^−1^ for ascorbic acid, 60 μg ml^−1^ for AE and 97 μg ml^−1^ for HE. In order to improve antioxidant background, superoxide radical scavenger activity was reached by xanthine/xanthine oxidase system (Figure 2B), where IC_50_ values were 1 μg ml^−1^, 9 μg ml^−1^ and 127 μg ml^−1^ for ascorbic acid, AE and HE, respectively.

Additionally, ORAC and FRAP assays fulfilled the antioxidant profile of hemp extracts. AE demonstrated 0.91 μmol TE/g sample and 17.75 μmol Fe^2+^/g extract, respectively, while HE showed 7.37 μmol TE/g sample and 21.94 μmol Fe^2+^/g extract, respectively.

### 3.4 Cell culture

#### 3.4.1 MTT assay

**FIGURE 3:**
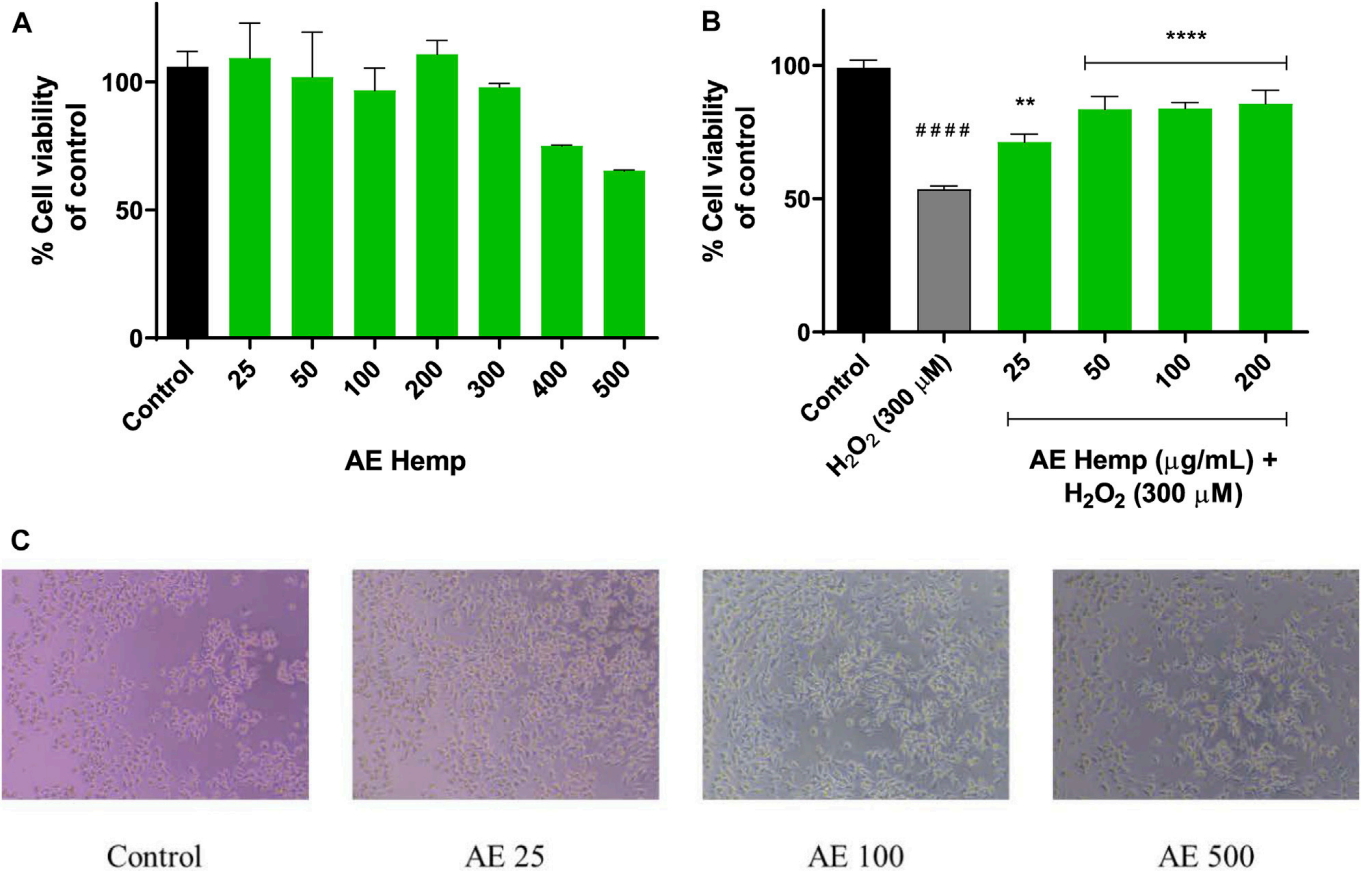
Mitochondrial response. **(A)** Cytotoxicity of Neuro-2a cells after exposure to different concentrations of *Cannabis sativa* aqueous extract. **(B)** Cytoprotective effects of *Cannabis sativa* aqueous extract *versus* hydrogen peroxide (300 µM). **(C)** Inverted microscope images of Neuro-2a cells (0–500 μg/ml) Note: **p < 0.01 versus H_2_O_2_; ****p < 0.001 versus H_2_O_2_; ####*p* < 0.001 versus control.

Figure 3A shows the Neuro-2a mitochondrial response to AE. A vast range of concentrations was tested in these neurons. Lower concentrations of this polyphenolic extract (25–50 μg/ml) improved mitochondrial activity even more than control cells (Figure 3C). Higher concentrations such us 400 and 500 μg/ml started to decrease mitochondrial activity which is related to a reduction of neuronal viability, but no significant differences were achieved meaning that this extract is not cytotoxic. Same concentrations were taken for HE resulting in cytotoxicity. Results were expressed over control cells.

The subsequent purpose was aimed to determine the cytoprotective effect of the extract against a prooxidant agent (H_2_O_2_, 300 µM) on Neuro-2a cells. The concentrations with best results in the previous assay were selected for this aiming. As seen in Figure 3B, cotreatments (hemp extract dilutions and hydrogen peroxide) resulted in significant differences against hydrogen peroxide-treated (53%) cells by ameliorating cell viability. These concentrations (25–200 μg/ml) enhanced cell viability in 20–30% over insulted neurons.

#### 3.4.2 ROS production

**FIGURE 4:**
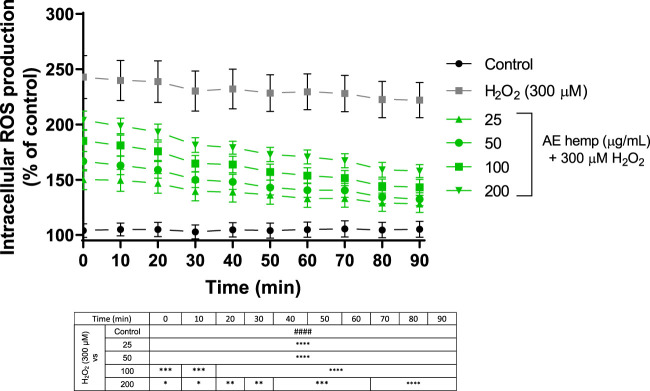
ROS production in Neuro-2a cells subjected to oxidative stress by hydrogen peroxide ((300 µM) and treatments with *Cannabis sativa* aqueous extract (25–200 μg/ml). Data are expressed as percentage over control cells and the assay was carried out for 90 min in order to measure intracellular ROS production. Note: ####*p* < 0.0001 *versus* control. Significant differences appeared at the starting point for H_2_O_2_-N2a cells over control cells. Since 0 min, 25 and 50 μg/ml pre-treatments were associated with significant differences (*p* < 0.0001). However, 100 and 200 μg/ml pre-treatments were associated with significant differences (*p* < 0.0005 and *p* < 0.05, respectively). From 70 min and above, highest significant differences were reached (*p* < 0.0001) for all treatments against hydrogen peroxide.

ROS detection confirmed the antioxidant potential of AE (Figure 4). In this 90 min assay, Neuro-2a cells were simultaneously exposed to different AE concentrations and hydrogen peroxide. Since T = 0, these dilutions countered the effect of the free radicals generated by the prooxidant. These reactive oxygen species produced by hydrogen peroxide treated cells maintained around 220–270% over control cells while cotreated cells (hemp + H_2_O_2_) reduced this production below 200%. Even more, last measurements continued to reduce ROS production almost reaching values of control cells.

## 4 Discussion

Oxidative stress has been identified as one of the main etiologies of neurodegenerative diseases and metabolic syndromes. Alzheimer’s disease (AD) and Parkinson’s disease (PD) are two disorders with the most prevalence in the world. Acetylcholine and dopamine are reduced in both diseases, respectively. As a matter of fact, the enzyme inhibition effects of *C. sativa* extracts on two important enzymes, AChE and MAO-A, were investigated and results are presented in Table 1.

Acetylcholine (ACh), a neurotransmitter of the cholinergic system, plays an important role in the treatment of numerous psychiatric disorders (Mineur et al., 2013). The enzyme acetylcholinesterase (AChE) deactivates neuronal impulse transmission by rapidly hydrolyzing acetylcholine in the cholinergic pathway in the peripheral and central nervous systems. For this reason, acetylcholinesterase inhibitors have historically been used for the treatment of various neurological pathologies, memory and cognitive impairment (Brady et al., 2011). The psychoactive effect of cannabis has been attributed to its main component, Δ9-THC, which is considered a potent inhibitor of AChE (Eubanks et al., 2006).

In the present study, the role of two hemp extracts was evaluated by an AChE inhibition assay. A dose-dependent relationship was observed (Figure 1A) with an IC_50_ value of 509 μg ml^−1^ and 640 μg ml^−1^ for AE and HE, respectively. The reference drug used in Alzheimer’s is galantamine, a substance that in this bioassay exhibited an IC_50_ of 1 μg ml^−1^. The extreme difference between the values of galantamine and *C. sativa* extracts indicated that cannabinoids exert dose-dependent activity, at high doses, for the inhibition of AChE. On the other hand, the work of Sugarman and collaborators reported that cannabinoids can cause inhibition of the release of acetylcholine, contributing to acute cognitive deficits by binding to the presynaptic CB1-R located in cholinergic nerve terminals (Sugarman et al., 2019). Taken together, these data suggest that cannabinoids act on the cholinergic system and raise the question of whether increasing acetylcholine levels pharmacologically can counteract the cognitive effects of cannabinoids by administering an acetylcholinesterase inhibitor such as galantamine or polyphenols. There is only an article related to luteolin-7-O-glucuronide, which is the major flavonoid of AE, and *Callicarpa maingayi*, although an IC_50_ value was not reached for AChE inhibition (Ado et al., 2019); this is because phytochemical composition plays a crucial role on this kind of bioassays.

MAOs are enzymes that play a key role in the degradation of exogenous and endogenous amines. The MAO family consists of two isoenzymes, MAO-A and MAO-B, which differ in their selectivity towards substrates and inhibitors. Although the two isoforms share great structure similarity, they diverge in the shape and volume of the catalytic site (Distinto et al., 2016).

MAO-A is an enzyme that catalyzes the oxidative deamination of a series of neurotransmitters, dietary amines, and xenobiotics. It is located in the outer mitochondrial membrane and preferentially oxidizes serotonin and norepinephrine and is inhibited by low concentrations of clorgyline (Cohen, 1983). Because of this, MAO-A inhibition can alleviate depression and provide protection against oxidative neurodegeneration, such as in Parkinson’s disease. Monoamine oxidase inhibitors (MAOIs) are one of the most important types of drugs prescribed for the treatment of depression, although they are a last-line treatment due to the risk of drug interaction with diet or other drugs (Olsen et al., 2008).

The IC_50_ results for this analysis were 315 μg ml^−1^ for AE, while HE was not able to reach the IC_50_. Barely any manuscript has been found on the MAO-A enzyme regarding *C. sativa*, an aqueous extract exhibited an IC_50_ value of 50.12 μg ml^−1^ (Rea et al., 2020), a slightly lower result than the one obtained in this project. It is noticed that CBD from hashish has no effect on the activity of MAO-A in brain tissues which determines why HE did not show any inhibitory effect on this enzyme (Chan and Duncan, 2021). In addition, cannabinoids diminish MAO activity at really high-level concentrations (Fišar, 2010).

The extracts of *C. sativa* showed antioxidant activity against the production of DPPH radicals, whose IC_50_ was 60 μg ml^−1^ for AE and 97 μg ml^−1^ for HE. Previous studies indicate that antioxidant activity is closely related to polyphenols, which have strong antioxidant activities. In them, a significant correlation was observed between the phenolic concentration and antioxidant activity evaluated by the DPPH method (Paja̧k et al., 2014). Luteolin-7-O-glucuronide and apigenin glucuronide were isolated from *Patrinia villosa* and IC_50_ values were 0.011 μg ml^−1^ in both cases (Liu et al., 2021), but for a CBD and Δ9-THC extract it was 141.9 μg ml^−1^ (Hacke et al., 2019).

The xanthine/xanthine oxidase assay was performed to measure the capacity of the extracts to scavenge superoxide radicals, triggering an IC_50_ of 9 μg ml^−1^ and 127 μg ml^−1^ for AE and HE, respectively. These results agree with those of the article on which this study is based, since the IC_50_s were 7.06 μg ml^−1^ and 63.84 μg ml^−1^ (Mazzara et al., 2022). Besides, the flavonoid luteolin-7-O-glucuronide obtained an IC_50_ of 0.24 μg ml^−1^ (Roza et al., 2016), another reason that support polyphenols from AE as excellent radical superoxide scavengers. No previous studies were found regarding apigenin glucuronide nor CBD and Δ9-THC.

The FRAP assay measures the antioxidant power through the reduction of the complex of ferric ions (Fe^3+^) to that of ferrous ions (Fe^2+^), acquiring an intense blue color produced by antioxidants in acid media and proportionally to these (Gulcin, 2020). The analysis of this test yielded results of 21.94 μmol Fe^2+^eq/g for HE and 17.75 μmol Fe^2+^eq/g for AE. A similar study analysed cannabis essential oil and CBD, with results of 12.9 μmol Fe^2+^eq/g and 10.8 μmol Fe^2+^eq/g (Benelli et al., 2017), respectively. From both studies, it can be deduced that the presence of CBD and other phytocannabinoids seemed to contribute significantly to the antioxidant activity of the cannabis extract and, depending on the harvest and cultivar conditions, physicochemical composition could alter antioxidant profile of the plants.

The ORAC assay measures the ability of antioxidants to absorb oxygen radicals, which can be easily quantified by fluorescence proportional to the breaking of oxygen chains as previously described. The results for this analysis were 0.91 μmol TE g^−1^ for AE and 7.37 μmol TE g^−1^ for HE. These values denote a much higher antioxidant capacity to neutralize peroxyl radicals by HE. Comparing with a study carried out by Teh et al., an extract of *C. sativa* achieved an ORAC result of 0.246 μmol TE g^−1^ (Teh et al., 2016), leading us to know that the phytocannabinoids of this cultivar have a great antioxidant power for the ORAC test, having achieved results almost four times higher for AE and up to 30 times higher for HE.

Not only the total content but also the type of phenolic compounds (chemical structure, number and position of the hydroxyl group, nature of the substitutions in the aromatic rings) perform a very important role in the antioxidant activity (Kolniak-Ostek, 2016). This would explain why AE had a lower IC_50_ in this study but also most of the solvents used in these assays were water soluble which may help to dissolve the extract better than HE which is less polar.

Polyphenols are natural compounds that have shown great antioxidant activity due to their structures and free radical scavenging capacity, but also owing to the modulation of other pathways such as endogenous antioxidant enzymes (Bors et al., 1990; Amić et al., 2007; Halliwell, 2012; Tsuji et al., 2013). As ROS levels decrease, the activity of antioxidant enzymes (superoxide dismutase, catalase, and glutathione peroxidase) increases. These endogenous enzymes can remove ROS more easily, showing a better response in cells that improve physiological antioxidant defense (Zhang and Tsao, 2016).

An imbalance between the production of reactive oxygen species (ROS) and the availability of adequate endogenous antioxidants can cause oxidative stress at the cellular level with detrimental effects on membranes, proteins, enzymes and DNA, leading to the progression of chronic diseases, inflammation and carcinogenesis (Girgih et al., 2013). The antioxidant effects of natural products could be considered as a first idea to detect their relevance and ethnopharmacological potential (Zengin et al., 2018). For this reason, the hemp polyphenolic extract (AE) was tested on a neuronal cell line (Neuro-2a). As this is a novel fraction from hemp extraction, no previous studies have been detected in literature on these neurons, but some studies related to luteolin and apigenin were found.

MTT assay revealed that the polar fraction of *C. sativa* is not cytotoxic, even more, it increased mitochondrial activity at low concentrations (25 and 50 μg/ml) confirmed by Figures 3A,C. Once these cells were exposed to a toxic agent such as hydrogen peroxide, the extract demonstrated a great neuroprotective effect on this radical producer neutralizing reactive oxygen species, significant differences were achieved in each concentration tested. The lowest concentration (25 μg/ml) showed less significant differences due to the concentration of the hydrogen peroxide was too high to counteract its action for that period although the rest of concentrations produced a greater mitochondrial response against the prooxidant. Mira et al. (2015) studied *Angelica shikokiana* impact on Neuro-2a exposed to H_2_O_2_. This manuscript determined that luteolin protected cells from neurotoxicity and it was able to scavenge intracellular ROS at 50 and 100 μM and presented significant differences. As shown in Figure 4, the four concentrations tested exhibited significant differences since the first moment. What stands out in this figure is the general pattern of the extract which decreases intracellular reactive oxygen species along the experiment versus the toxic agent. Indian researchers tested *Grewia tiliaefolia*, a rich source of flavonoids such as vitexin, on Neuro-2a subjected to glutamate (5 mM). The accumulation of this neurotransmitter is involved in Alzheimer’s Disease. *Grewia tiliaefolia* treatments (25–100 μg/ml) inhibited oxidative stress damage by reducing the production of intracellular ROS and cell viability was barely affected (Malar et al., 2018).

In conclusion, this study provided new insights into the biological activities of two different extracts of *C. sativa*. It was revealed that these extracts constitute a valuable and interesting natural source of bioactive molecules with great antioxidant properties, potentially capable of preventing neurodegenerative diseases. However, among the different bioassays, the results showed that these extracts are more effective in terms of antioxidant content.

It should be noted that both extracts showed a striking antioxidant capacity for the FRAP and ORAC tests, especially HE, and an interesting profile for the DPPH and superoxide radical scavenging tests, with AE especially standing out in the latter. Regarding the enzyme inhibition tests, polyphenols from AE showed superior response than phytocannabinoids from HE.

Finally, further studies are required to better understand the mechanism of action of these extracts as antioxidants and pharmacological agents. These results are very promising, considering every hemp derivative essential for industrial and technological development in the food, pharmaceutical and cosmetic industries.

## Data Availability

The raw data supporting the conclusions of this article will be made available by the authors, without undue reservation.

## References

[B1] Ado M. A. Maulidiani M. Ismail I. S. Ghazali H. M. Shaari K. Abas F. (2019). Acetylcholinesterase and α-glucosidase inhibitory compounds from Callicarpa maingayi. Nat. Prod. Res 35, 2992–2996. 10.1080/14786419.2019.1679138 31631709

[B2] Akinrinde A. S. Adebiyi O. E. (2019). Neuroprotection by luteolin and gallic acid against cobalt chloride-induced behavioural, morphological and neurochemical alterations in Wistar rats. Neurotoxicology 74, 252–263. 10.1016/J.NEURO.2019.07.005 31362009

[B3] Amić D. Davidović-Amić D. Beslo D. Rastija V. Lucić B. Trinajstić N. (2007). SAR and QSAR of the antioxidant activity of flavonoids. Curr. Med. Chem. 14, 827–845. 10.2174/092986707780090954 Accessed May 31, 2018) 17346166

[B4] Andre C. M. Hausman J. F. Guerriero G. (2016). Cannabis sativa: The plant of the thousand and one molecules. Front. Plant Sci. 7, 19. 10.3389/fpls.2016.00019 26870049 PMC4740396

[B5] Atalay S. Jarocka-karpowicz I. Skrzydlewskas E. (2019). Antioxidative and anti-inflammatory properties of cannabidiol. Antioxidants 9, 21. 10.3390/ANTIOX9010021 PMC7023045 31881765

[B6] Bautista J. L. Yu S. Tian L. (2021). Flavonoids in cannabis sativa: Biosynthesis, bioactivities, and biotechnology. ACS Omega, 6, 5119–5123. 10.1021/ACSOMEGA.1C00318/ASSET/IMAGES/LARGE/AO1C00318_0001 33681553 PMC7931196

[B7] Benelli G. Pavela R. Lupidi G. Nabissi M. Petrelli R. Ngahang Kamte S. L. (2017). The crop-residue of fiber hemp cv. Futura 75: From a waste product to a source of botanical insecticides. Environ. Sci. Pollut. Res. Int. 25, 10515–10525. 10.1007/S11356-017-0635-5 29105041

[B8] Bors W. Heller W. Michel C. Saran M. (1990). Flavonoids as antioxidants: Determination of radical-scavenging efficiencies. Methods Enzymol. 186, 343–355. 10.1016/0076-6879(90)86128-I 2172711

[B9] Brady K. T. Gray K. M. Tolliver B. K. (2011). Cognitive enhancers in the treatment of substance use disorders: Clinical evidence. Pharmacol. Biochem. Behav. 99, 285–294. 10.1016/J.PBB.2011.04.017 21557964 PMC3114106

[B10] Campos A. C. Fogaça M. V. Sonego A. B. Guimarães F. S. (2016). Cannabidiol, neuroprotection and neuropsychiatric disorders. Pharmacol. Res. 112, 119–127. 10.1016/J.PHRS.2016.01.033 26845349

[B11] Cermak R. (2007). Effect of dietary flavonoids on pathways involved in drug metabolism. Expert. Opin. Drug. Metab. Toxic. 4, 17–35. 10.1517/17425255.4.1.17 18370856

[B12] Chan J. Z. Duncan R. E. (2021). Regulatory effects of cannabidiol on mitochondrial functions: A review. Cells 10, 1251. 10.3390/CELLS10051251 34069407 PMC8159073

[B13] Chandrasekhar Y. Phani Kumar G. Ramya E. M. Anilakumar K. R. (2018). Gallic acid protects 6-OHDA induced neurotoxicity by attenuating oxidative stress in human dopaminergic cell line. Neurochem. Res. 43, 1150–1160. 10.1007/s11064-018-2530-y 29671234

[B14] Chen G. Luo J. (2010). Anthocyanins: Are they beneficial in treating ethanol neurotoxicity? Neurotox. Res. 17, 91–101. 10.1007/s12640-009-9083-4 19590929 PMC4992359

[B15] Cohen G. (1983). The pathobiology of Parkinson’s disease: Biochemical aspects of dopamine neuron senescence. J. Neural transm. Suppl. 19, 89–103. Available at: https://pubmed.ncbi.nlm.nih.gov/6321651/ (Accessed July 2, 2022). 6321651

[B16] Dávalos A. Gómez-Cordovés C. Bartolomé B. (2004). Extending applicability of the oxygen radical absorbance capacity (ORAC-Fluorescein) assay. J. Agric. Food Chem. 52, 48–54. 10.1021/jf0305231 14709012

[B17] Distinto S. Meleddu R. Yanez M. Cirilli R. Bianco G. Sanna M. L. (2016). Drug design, synthesis, *in vitro* and *in silico* evaluation of selective monoaminoxidase B inhibitors based on 3-acetyl-2-dichlorophenyl-5-aryl-2, 3-dihydro-1, 3, 4-oxadiazole chemical scaffold. Eur. J. Med. Chem. 108, 542–552. 10.1016/J.EJMECH.2015.12.026 26717204

[B18] Eubanks L. M. Rogers C. J. Beuscher IV A. E. Koob G. F. Olson A. J. Dickerson T. J. (2006). A molecular link between the active component of marijuana and Alzheimer’s disease pathology. Mol. Pharm. 3, 773–777. 10.1021/MP060066M/ASSET/IMAGES/MEDIUM/MP060066MN00001 17140265 PMC2562334

[B19] Fernández-Moriano C. González-Burgos E. Divakar P. K. Crespo A. Gómez-Serranillos M. P. (2016). Evaluation of the antioxidant capacities and cytotoxic effects of ten parmeliaceae lichen species. Evid. Based. Complement. Altern. Med. 201, 3169751. 10.1155/2016/3169751 PMC5203883 28074101

[B20] Fišar Z. (2010). Inhibition of monoamine oxidase activity by cannabinoids. Naunyn. Schmiedeb. Arch. Pharmacol. 381, 563–572. 10.1007/S00210-010-0517-6 20401651

[B21] Girgih A. T. Alashi A. He R. Malomo S. Aluko R. E. (2013). Preventive and treatment effects of a hemp seed (Cannabis sativa L.) meal protein hydrolysate against high blood pressure in spontaneously hypertensive rats. Eur. J. Nutr. 53, 1237–1246. 10.1007/S00394-013-0625-4 24292743

[B22] Gulcin İ. (2020). Antioxidants and antioxidant methods: An updated overview. Arch. Toxicol. 94, 651–715. 10.1007/S00204-020-02689-3 32180036

[B23] Hacke A. C. M. Lima D. De Costa F. Deshmukh K. Li N. Chow A. M. (2019). Probing the antioxidant activity of Δ9-tetrahydrocannabinol and cannabidiol in Cannabis sativa extracts. Analyst 144, 4952–4961. 10.1039/C9AN00890J 31318364

[B24] Halliwell B. (2012). Free radicals and antioxidants: Updating a personal view. Nutr. Rev. 70, 257–265. 10.1111/j.1753-4887.2012.00476.x 22537212

[B25] Ibeas Bih C. Chen T. Nunn A. V. W. Bazelot M. Dallas M. Whalley B. J. (2015). Molecular targets of cannabidiol in neurological disorders. Neurotherapeutics. 12, 699–730. 10.1007/S13311-015-0377-3 26264914 PMC4604182

[B26] Junior N. C. F. dos- Santos-Pereira M. Guimarães F. S. Del Bel E. (2019). Cannabidiol and cannabinoid compounds as potential strategies for treating Parkinson’s disease and l-DOPA-induced dyskinesia. Neurotox. Res. 37, 12–29. 10.1007/S12640-019-00109-8 31637586

[B27] Kolniak-Ostek J. (2016). Content of bioactive compounds and antioxidant capacity in skin tissues of pear. J. Funct. Foods 23, 40–51. 10.1016/J.JFF.2016.02.022

[B28] Laun A. S. Shrader S. H. Brown K. J. Song Z. H. (2018). GPR3, GPR6, and GPR12 as novel molecular targets: Their biological functions and interaction with cannabidiol. Acta Pharmacol. Sin. 40, 300–308. 10.1038/s41401-018-0031-9 29941868 PMC6460361

[B29] LeBel C. P. Ischiropoulos H. Bondy S. C. (1992). Evaluation of the probe 2’, 7’-dichlorofluorescin as an indicator of reactive oxygen species formation and oxidative stress. Chem. Res. Toxicol. 5, 227–231. 10.1021/tx00026a012 1322737

[B30] Li H. Liu Y. Tian D. Tian L. Ju X. Qi L. (2020). Overview of cannabidiol (CBD) and its analogues: Structures, biological activities, and neuroprotective mechanisms in epilepsy and Alzheimer’s disease. Eur. J. Med. Chem. 192, 112163. 10.1016/J.EJMECH.2020.112163 32109623

[B31] Liu Y. C. Sui N. Wang J. Q. Wang X. Liu W. Xiang Z. (2021). A novel flavonoid with antioxidant activity from *Patrinia villosa* (Thunb.) Juss. Nat. Prod. Res. 36, 2977–2983. 10.1080/14786419.2021.1935931 34085576

[B32] López V. Akerreta S. Casanova E. García-Mina J. M. Cavero R. Y. Calvo M. I. (2007). *In vitro* antioxidant and anti-rhizopus activities of lamiaceae herbal extracts. Plant Foods Hum. Nutr. 62, 151–155. 10.1007/s11130-007-0056-6 17912643

[B33] Malar D. S. Prasanth M. I. Shafreen R. B. Balamurugan K. Devi K. P. (2018). Grewia tiliaefolia and its active compound vitexin regulate the expression of glutamate transporters and protect Neuro-2a cells from glutamate toxicity. Life Sci. 203, 233–241. 10.1016/J.LFS.2018.04.047 29704481

[B34] Mazzara E. Carletti R. Petrelli R. Mustafa A. M. Caprioli G. Fiorini D. (2022). Green extraction of hemp (cannabis sativa L.) using microwave method for recovery of three valuable fractions (essential oil, phenolic compounds and cannabinoids): A central composite design optimization study. J. Sci. Food Agric. 10.1002/JSFA.11971 PMC9790304 35485728

[B35] Mineur Y. S. Obayemi A. Wigestrand M. B. Fote G. M. Calarco C. A. Li A. M. (2013). Cholinergic signaling in the hippocampus regulates social stress resilience and anxiety- and depression-like behavior. Proc. Natl. Acad. Sci. U. S. A. 110, 3573–3578. 10.1073/PNAS.1219731110/SUPPL_FILE/PNAS.201219731SI 23401542 PMC3587265

[B36] Mira A. Yamashita S. Katakura Y. Shimizu K. (2015). *In vitro* neuroprotective activities of compounds from Angelica shikokiana Makino. Molecules 20, 4813–4832. 10.3390/MOLECULES20034813 25786165 PMC6272295

[B37] Nabavi S. F. Khan H. D’onofrio G. Šamec D. Shirooie S. Dehpour A. R. (2018). Apigenin as neuroprotective agent: Of mice and men. Pharmacol. Res. 128, 359–365. 10.1016/J.PHRS.2017.10.008 29055745

[B38] Olsen H. T. Stafford G. I. van Staden J. Christensen S. B. Jäger A. K. (2008). Isolation of the MAO-inhibitor naringenin from Mentha aquatica L. J. Ethnopharmacol. 117, 500–502. 10.1016/j.jep.2008.02.015 18372132

[B39] Paja̧k P. Socha R. Gałkowska D. Roz̊nowski J. Fortuna T. (2014). Phenolic profile and antioxidant activity in selected seeds and sprouts. Food Chem. 143, 300–306. 10.1016/J.FOODCHEM.2013.07.064 24054243

[B40] Pellati F. Borgonetti V. Brighenti V. Biagi M. Benvenuti S. Corsi L. (2018). Cannabis sativa L. And nonpsychoactive cannabinoids: Their chemistry and role against oxidative stress, inflammation, and cancer. Biomed. Res. Int. 2018, 1691428, 10.1155/2018/1691428 30627539 PMC6304621

[B41] Peñarrieta J. M. Tejeda L. Mollinedo P. Vila J. L. Bravo J. A. (2014). Phenolic compounds in food. Boliv. J. Chem. 31, 68

[B42] Peng B. Yang Q. Joshi R. B. Liu Y. Akbar M. Song B. J. (2020). Role of alcohol drinking in Alzheimer’s disease, Parkinson’s disease, and amyotrophic lateral sclerosis. Int. J. Mol. Sci. 202021, 2316. 10.3390/IJMS21072316 PMC7177420 32230811

[B43] Pollastro F. Minassi A. Fresu L. G. (2017). Cannabis phenolics and their bioactivities. Curr. Med. Chem. 25, 1160–1185. 10.2174/0929867324666170810164636 28799497

[B44] Rea J. García-Giménez M. D. Santiago M. De la Puerta R. Fernández-Arche M. A. (2020). Hydroxycinnamic acid derivatives isolated from hempseed and their effects on central nervous system enzymes. Int J Food Sci Nutr 72, 184. 10.1080/09637486.2020.1793305 32664762

[B45] Rhee I. K. van de Meent M. Ingkaninan K. Verpoorte R. (2001). Screening for acetylcholinesterase inhibitors from Amaryllidaceae using silica gel thin-layer chromatography in combination with bioactivity staining. J. Chromatogr. A. 915, 217–223. Available at: http://www.ncbi.nlm.nih.gov/pubmed/11358251 (Accessed February 12, 2018). 11358251 10.1016/s0021-9673(01)00624-0

[B46] Rodríguez-Chávez J. L. Coballase-Urrutia E. Nieto-Camacho A. Delgado-Lamas G. (2015). Antioxidant capacity of “mexican arnica” heterotheca inuloides cass natural products and some derivatives: Their anti-inflammatory evaluation and effect on *C. elegans* life span. Oxid. Med. Cell. Longev. 2015, 843237. 10.1155/2015/843237 25821555 PMC4363644

[B47] Rodriguez-Martin N. M. Toscano R. Villanueva A. Pedroche J. Millan F. Montserrat-De La Paz S. (2019). Neuroprotective protein hydrolysates from hemp (Cannabis sativa L.) seeds. Food Funct. 10, 6732–6739. 10.1039/C9FO01904A 31576391

[B48] Roza O. Martins A. Hohmann J. Lai W. C. Eloff J. Chang F. R. (2016). Flavonoids from cyclopia genistoides and their xanthine oxidase inhibitory activity. Planta Med. 82, 1274–1278. 10.1055/S-0042-110656 27392243

[B49] Russo E. B. Jiang H. E. Li X. Sutton A. Carboni A. Del Bianco F. (2008). Phytochemical and genetic analyses of ancient cannabis from Central Asia. J. Exp. Bot. 59, 4171–4182. 10.1093/JXB/ERN260 19036842 PMC2639026

[B50] Sirikantaramas S. Taura F. (2017). Cannabinoids: Biosynthesis and biotechnological applications. Cannabis sativa l. - Bot. Biotechnol. 8, 183–206. 10.1007/978-3-319-54564-6_8/COVER/

[B51] Sugarman D. E. De Aquino J. P. Poling J. Sofuoglu M. (2019). Feasibility and effects of galantamine on cognition in humans with cannabis use disorder. Pharmacol. Biochem. Behav. 181, 86–92. 10.1016/J.PBB.2019.05.004 31082417 PMC6545124

[B52] Teh S. S. Bekhit A. E. D. A. Carne A. Birch J. (2016). Antioxidant and ACE-inhibitory activities of hemp (Cannabis sativa L.) protein hydrolysates produced by the proteases AFP, HT, Pro-G, actinidin and zingibain. Food Chem. 203, 199–206. 10.1016/J.FOODCHEM.2016.02.057 26948606

[B53] Tsuji P. A. Stephenson K. K. Wade K. L. Liu H. Fahey J. W. (2013). Structure-activity analysis of flavonoids: Direct and indirect antioxidant, and antiinflammatory potencies and toxicities. Nutr. Cancer 65, 1014–1025. 10.1080/01635581.2013.809127 24087992

[B54] Whiting P. F. Wolff R. F. Deshpande S. Di Nisio M. Duffy S. Hernandez A. V. (2015). Cannabinoids for medical use: A systematic review and meta-analysis. JAMA 313, 2456–2473. 10.1001/JAMA.2015.6358 26103030

[B55] Zengin G. Menghini L. Sotto A. D. Mancinelli R. Sisto F. Carradori S. (2018). Chromatographic analyses, *in vitro* biological activities, and cytotoxicity of cannabis sativa L. Essential oil: A multidisciplinary study. Molecules 23, 3266. 10.3390/MOLECULES23123266 PMC6320915 30544765

[B56] Zhang H. Tsao R. (2016). Dietary polyphenols, oxidative stress and antioxidant and anti-inflammatory effects. Curr. Opin. Food Sci. 8, 33–42. 10.1016/j.cofs.2016.02.002

[B57] Zhang Z. Li G. Szeto S. S. W. Chong C. M. Quan Q. Huang C. (2015). Examining the neuroprotective effects of protocatechuic acid and chrysin on *in vitro* and *in vivo* models of Parkinson disease. Free Radic. Biol. Med. 84, 331–343. 10.1016/j.freeradbiomed.2015.02.030 25769424

